# Histone N-alpha terminal modifications: genome regulation at the tip of the tail

**DOI:** 10.1186/s13072-020-00352-w

**Published:** 2020-07-17

**Authors:** Christina Demetriadou, Costas Koufaris, Antonis Kirmizis

**Affiliations:** Epigenetics Laboratory, Department of Biological Sciences, University of Cyprus, 2109 Nicosia, Cyprus

**Keywords:** Histone modifications, Histone N-terminal transferases, N-terminal acetyltransferases, N-terminal methyltransferases, NAA40, NTMT1

## Abstract

Histone proteins are decorated with numerous post-(PTMs) or co-(CTMs) translational modifications mainly on their unstructured tails, but also on their globular domain. For many decades research on histone modifications has been focused almost solely on the biological role of modifications occurring at the side-chain of internal amino acid residues. In contrast, modifications on the terminal N-alpha amino group of histones—despite being highly abundant and evolutionarily conserved—have been largely overlooked. This oversight has been due to the fact that these marks were being considered inert until recently, serving no regulatory functions. However, during the past few years accumulating evidence has drawn attention towards the importance of chemical marks added at the very N-terminal tip of histones and unveiled their role in key biological processes including aging and carcinogenesis. Further elucidation of the molecular mechanisms through which these modifications are regulated and by which they act to influence chromatin dynamics and DNA-based processes like transcription is expected to enlighten our understanding of their emerging role in controlling cellular physiology and contribution to human disease. In this review, we clarify the difference between N-alpha terminal (Nt) and internal (In) histone modifications; provide an overview of the different types of known histone Nt-marks and the associated histone N-terminal transferases (NTTs); and explore how they function to shape gene expression, chromatin architecture and cellular phenotypes.

## Introduction

Within the nucleus of eukaryotic cells, DNA is packaged into a highly organized chromatin structure. The building blocks of chromatin, known as nucleosomes, each comprises of 147 base pairs of DNA wrapped around an octameric protein complex which involves two copies of each of the four histone proteins, H3, H4, H2A and H2B. Histones are subjected to a wide variety of modifications by the addition of chemical groups both on their globular domain and at the N-terminal tails projecting from the nucleosomal core particle [1, 2]. The covalent addition of these functional groups on histone proteins occur either during (co-translationally) or after (post-translationally) their synthesis. Histone modifications represent an essential cellular mechanism in governing fundamental biological processes through the tight regulation of chromatin structure and gene expression. Acetylation and methylation are among the most well studied histone marks, whereas during the last few years novel modifications such as propionylation [3], serotonylation [4], glutarylation [5], and acylations [6] among others have been discovered. Generally, post-(PTMs) and co-translational (CTMs) modifications are directed by the function of three different group of specialized proteins: the “writers” which catalyze the addition of specific modifications, the “erasers” which are responsible for the removal of those modifications and the “readers” which recognize and interpret modifications to translate them into a specific functional outcome [7, 8].

There already exist several excellent reviews on the biological mechanisms and function of PTMs on histone tails or nucleosome core, e.g., [1, 2, 7–10]. These and other review papers reflect the primary focus of the epigenetics field on PTMs of internal histone amino acid residues, especially acetylation or methylation occurring at the side-chain amine groups. However, much less attention has until recently been given to the PTMs/CTMs on the N-alpha amino group at the *terminal* tip of histone proteins. The role of these terminal marks has been originally associated with protein stability since they were found to protect proteins from degradation [11]. The development of new mass spectrometry (MS) based proteomics methods that allow a more reliable detection and quantification of the histone N-alpha terminal modifications, coupled with new information on their important biological and physiological functions, have led to a radical reappraisal of these histone marks and of their biological significance.

This review will specifically examine the biology and regulatory functions of histone N-alpha terminal modifications, highlighting what has been recently unveiled and the important open questions.

## Histone N-alpha terminal vs internal modifications: what is the difference?

**Fig. 1:**
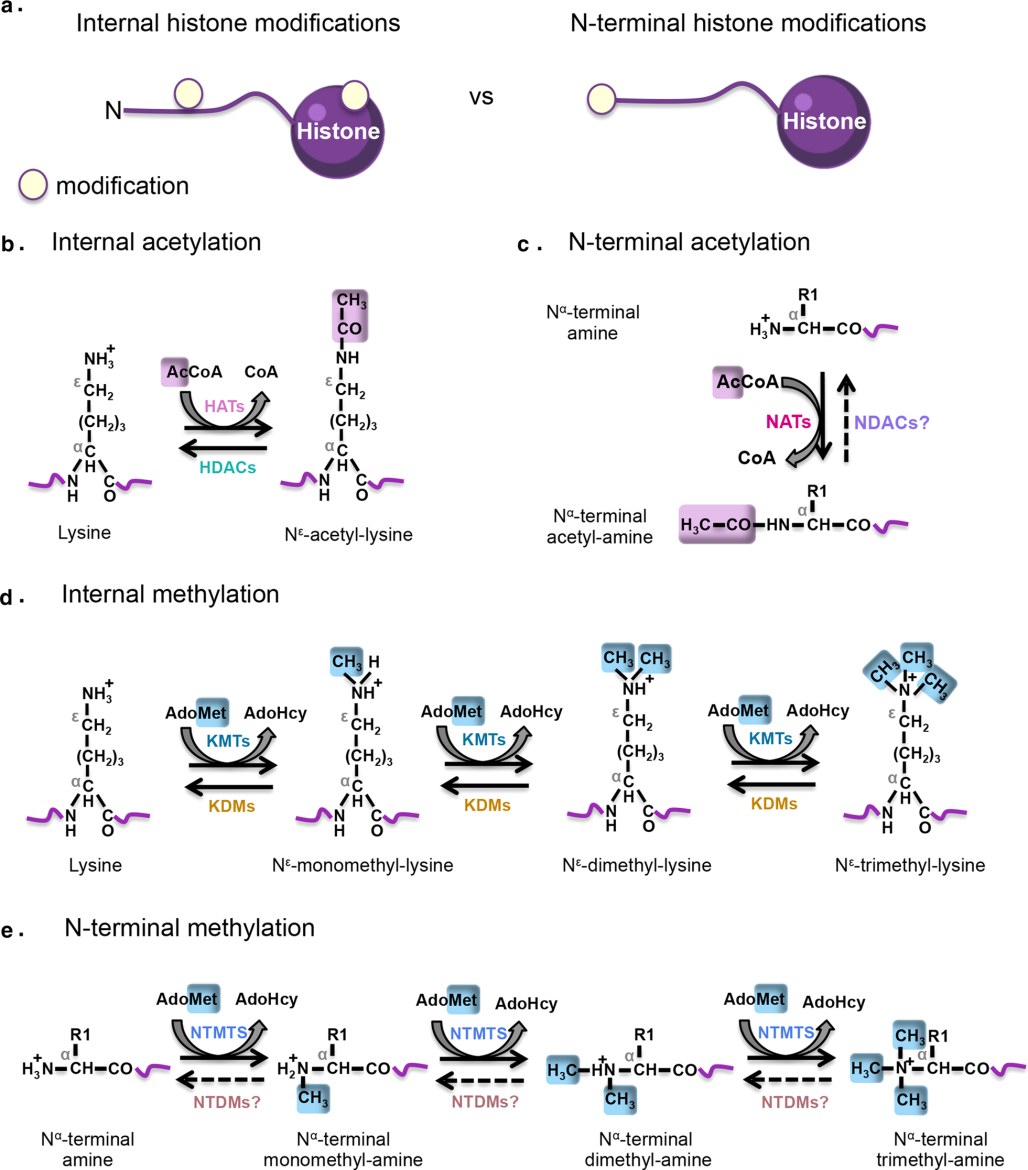
Comparison of Internal vs N-terminal histone modifications. **a** Internal (In) modifications are deposited on the side chain of internal amino acids on the octameric core domain or the N-terminal tails of histone proteins. Conversely, N-alpha terminal (Nt) marks are located at the N-terminal end of histone tails. **b** A HAT catalyzes the transfer of an acetyl group from Ac-CoA to the ε-amino group of an internal lysine residue. N^ε^-lysine acetylation is reversed by HDACs. **c** NATs transfer an acetyl group from Ac-CoA to the α-amino group of the first amino acid residue at the N-terminal tip of histone proteins. N-alpha terminal acetylation is believed to be static as no NDACs have been identified yet. **d** A variety of In-me are currently known to occur on different residues including lysine, arginine, and glutamine. As an example the mono-, di- or trimethylation of internal lysine ε-amino groups is catalyzed by KMTs using SAM as the methyl donor. The lysine demethylase reaction is driven by KDMs (LSD1 and JmjC domain-containing proteins). **e** The mono-, di- and trimethylation of the α-amino group on the first amino acid residue of histones is catalyzed by NTMTs using SAM as the methyl donor. N-alpha terminal methylation is described as a constitutive PTM as NTDMs remain to be discovered

Despite the variety of histone covalent modifications discovered thus far, acetylation and methylation are the most well documented. Internal (In) modifications occur on the side chain of internal amino acid residues (i.e., lysines, arginines, glutamines) on histone proteins, whereas N-alpha terminal (Nt) marks are located at the N-terminal end of histone tails (Fig. 1a). Importantly, the deposition of In and Nt histone modifications occurs through distinct sets of enzymes specialized for these tasks (Fig. 1b–e).

The In-acetylation (In-ac) comprises the covalent attachment of an acetyl group from acetyl-coenzyme A (Ac-CoA) to the epsilon-amino group (N^ε^) of an internal lysine residue, a process regulated by the opposing catalytic roles of histone acetyltransferases (HATs) and histone deacetylases (HDACs) (Fig. 1b) [12, 13]. Internal lysine acetylation on histone proteins is generally associated with transcriptional activation [7].

The In-methylation (In-me) is commonly deposited on lysine or arginine residues by histone methyltransferases. Within this group of enzymes, protein lysine methyltransferases (KMTs), transfer methyl groups from S-adenosyl methionine (SAM) to the epsilon (Ν^ε^) amino group of internal lysine residues (Fig. 1d). Each individual lysine residue can be mono-(N^ε^-monomethyl-lysine), di-(N^ε^-dimethyl-lysine), or trimethylated (N^ε^-trimethyl-lysine) (Fig. 1d). On the other hand, the guanidino (N^G^) amino group of internal arginine residues is monomethylated (N^G^-monomethyl-l-arginine) or asymmetrically (N^G^, N^G^-dimethyl-l-arginine)/symmetrically (N^G^, N′^G^-dimethyl-l-arginine) dimethylated by arginine methyltransferases (PRMTs) [7]. Interestingly, a new type of In-me has been discovered, which involves methylation on the side chain of an internal glutamine residue found within the core domain of histone H2A and it is catalyzed by fibrillarin [14]. Generally, the function of histone In-methylation is more complex than In-acetylation since it specifies both active and repressed chromatin states depending on the histone amino acid residue bearing the modification as well as, the degree of methylation [8]. For instance, H3K4me3, H3K79me2 and H3R17me2a are associated with transcriptional activation while H3K27me3, H4K20me2 and H3R2me2a are linked to gene silencing [8, 15]. In-methylation on lysine residues can be reversed by demethylases (KDMs) which are categorized into two enzymatic groups: the FAD-dependent and the JmjC domain containing histone lysine demethylases. Although arginine demethylases remain to be identified, there is supporting evidence that arginine demethylation can be catalyzed by the JmjC enzymes [16].

In contrast to In-modifications, Nt-marks occur on the alpha-amino group (N^α^) of the first amino acid residue of the histone proteins mainly upon the removal of the initiator N-terminal methionine (iNt-Met) by Met-aminopeptidases (MetAPs). These Nt-modifications occur through the action of histone N-terminal transferases (NTTs) which can establish the acetylation and methylation of the α-amino group. Nt-acetylation (Nt-ac) of histones involves the transfer of an acetyl moiety from Ac-CoA to the free α-amino group of the first amino acid residue on nascent polypeptides through the action of N-terminal acetyltransferases (NATs), a highly conserved family of enzymes that modify the N-terminal end of the majority of eukaryotic proteins (Fig. 1c); whereas, Nt-methylation (Nt-me) involves the addition of 1–3 methyl groups from SAM to the α-amino group on the very N-terminal tip of histones, and is specifically catalyzed by N-terminal methyltransferases (NTMTs) (Fig. 1e). Unlike the highly dynamic In-modifications, Nt-acetylation and Nt-methylation marks are so far believed to be static since neither N-terminal deacetylases (NDACS) nor N-terminal demethylases (NTDMs) have to our knowledge been discovered yet. Furthermore, the precise role of Nt-ac and Nt-me as activating and/or silencing marks is still under investigation.

It is important here to clarify that confusion can arise due to the frequent references in the literature to “N-terminal tail modifications” which in fact refers to Internal marks occurring at the first few amino acids within the histone tails, and not to the actual N-alpha terminal modifications. It is therefore critical to be careful both when using and reading the term N-terminal tail modifications.

## Histone N-alpha terminal acetylation

The N-alpha terminal acetylation (Nt-ac) of histone proteins has been reproducibly detected so far on H4, H2A, H2B, and H1. Nt-acetylation of H4 and H2A can be grouped together as these two histones are modified by the same NAT, N-alpha-acetyltransferase 40 (NAA40) [17]. The Nt-acetylation of histones H1 and H2B are distinct in that they are not deposited through NAA40—but possibly through other members of the NAT family - and potentially have distinct regulation and functions.

### Nt-acetylation of histones H4 and H2A via NAA40

Nt-acetylation of histones H4 (N-acH4) and H2A (N-acH2A) is an evolutionarily conserved modification from yeast to humans [17]. The addition of the acetyl moiety on H4/H2A neutralizes the positively charged free α-amino group, thus inhibiting ionization and other modifications to occur at the N-terminus and constructing a larger more hydrophobic N^α^-terminal serine residue. Proteomic investigations of PTMs/CTMs on histones H4 and H2A through MS-based methods have consistently revealed these modifications to be perhaps the most abundant marks of histone proteins. Examining mouse brains using an electron transfer dissociation MS method it was estimated that Nt-acetylation of H2A is at 87% and of H4 at 93%. In the same samples Nt-acetylation was also detected in 58% of canonical H1, but not for H2B or H3 peptides [18]. Independently, the group of Nicolas Young has found very high levels of N-acH4 in human cancer cells, 97-98% in SUM159 and MCF7 breast cancer cell lines [19, 20].

The common factor underlying Nt-acetylation on histones H4 and H2A is the specific catalysis of this modification by the NAT family member NAA40 [21]. Classically the NATs were considered to act co-translationally and to be highly promiscuous in the number of proteins they modify. For instance, the NatA and NatB complexes target an estimated 38% and 21% of the human proteome, respectively [21]. More recently, examples have emerged of NATs which are more selective in their targeting. An interesting case is NAA80 that acts selectively on Actin [22]. Similarly NAA40 is believed to specifically act on H4 and H2A [17, 21]. This specificity of NAA40 for these two histone proteins possibly arises from its exclusive recognition of the N-terminal sequence Ser(1)-Gly(2)-Arg(3)-Gly(4) (SGRG), found at the beginning of H4 and H2A [17]. Besides H4 and H2A, this recognition motif is also present at the N-terminal tail of the histone variant H2A.X that potentially could be subjected to Nt-ac by NAA40, but this has not been experimentally tested yet. Acetylation of the NAA40 substrate proteins is mediated through the GCN5-related N-acetyltransferase (GNAT) domain consisting of the conserved Arg/Gln-X-X-Gly-X-Gly/Ala Ac-CoA binding motif. Due to its structural divergence compared to other NATs, NAA40 does not require an accessory protein in order to function [21]. Instead, the unique N-terminal segment of NAA40 is necessary to stabilize the catalytic core domain, whereas its target specificity is achieved through direct interactions between serine 1 (S1) and arginine 3 (R3) residues on the histone proteins [23].

While most of the HAT enzymes modify histone proteins post-translationally, NAA40 is predominately localized in the cytosol where it binds to ribosomes and thought to catalyze N-acH4/H2A in a co-translational manner. However, immunofluorescence and biochemical fractionation experiments indicate that NAA40 can be also found in non-ribosomal forms within the nucleus [17]. This observation signifies that NAA40 may catalyze histone Nt-ac post-translationally as well. In fact, post-translational modifying activity has been already reported for other NAT family members, e.g., NAA80 was shown to N-alpha terminally acetylate Actin post-translationally in the cytosol [22], while NAA70 post-translationally modifies the N-alpha terminus of proteins in the chloroplast lumen [24]. For NAA40 it remains to be seen if it also acts post-translationally.

### The dynamic nature and biological functions of H4 Nt-acetylation

**Fig. 2:**
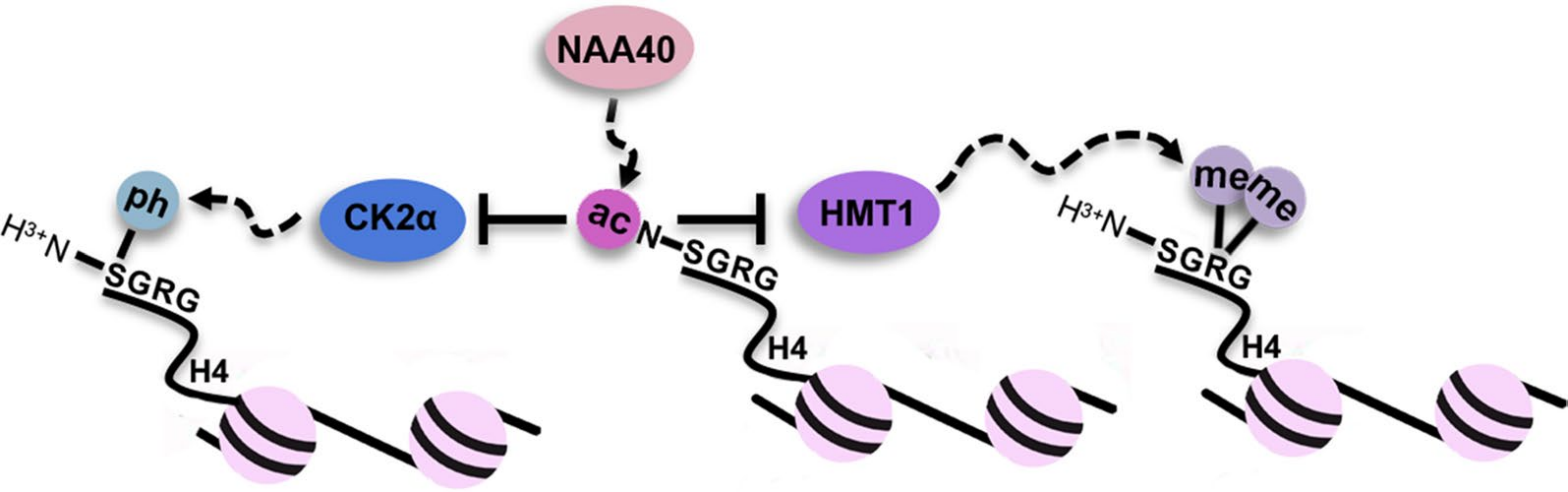
Interplay between N-terminal and Internal histone  modifications. N-terminal acetylation on histone H4 (N-acH4) blocks CK2α-mediated serine 1 (S1) phosphorylation [28]. Moreover, NAA40-mediated N-acH4 also inhibits the activity of HMT1 towards H4R3me2a [25]

Over the past few years work originating from our group and others has revealed that Nt-ac of histone H4 (N-acH4) is a dynamic modification, which influences important biological conditions such as cancer and longevity. Our group first postulated a role of N-acH4 in the control of ribosomal RNA expression and response to caloric restriction [25, 26]. Deletion of the yeast homolog of NAA40 (Nat4) and reduced deposition of N-acH4 induces Hmt1 methyltransferase to asymmetrically dimethylate histone H4 arginine 3 residue (H4R3me2a) leading to increased silencing of ribosomal DNA genes [25] (Fig. 2). In a subsequent study, we reported that caloric restriction (CR) in yeast suppresses Nat4 expression and the associated loss of N-acH4 is an essential step in promoting the longevity effects of CR. In fact, constitutive over-expression of Nat4 was sufficient to reduce the lifespan extension associated with CR, and deletion of the enzyme mimicked caloric restriction at the transcriptomic level [26]. Functionally, it was shown that Nat4 and its corresponding N-acH4 control the CR-mediated longevity pathway by negatively regulating the expression of key metabolic and stress-response genes through crosstalk with methylation at the adjacent H4R3 residue. Of note, another study in mouse models has linked NAA40 to hepatic lipid metabolism and aging [27]. In particular, NAA40 liver-specific knockout mice exhibit reduced fat mass, aberrant lipid metabolism and are protected from age-associated hepatic steatosis.

**Fig. 3:**
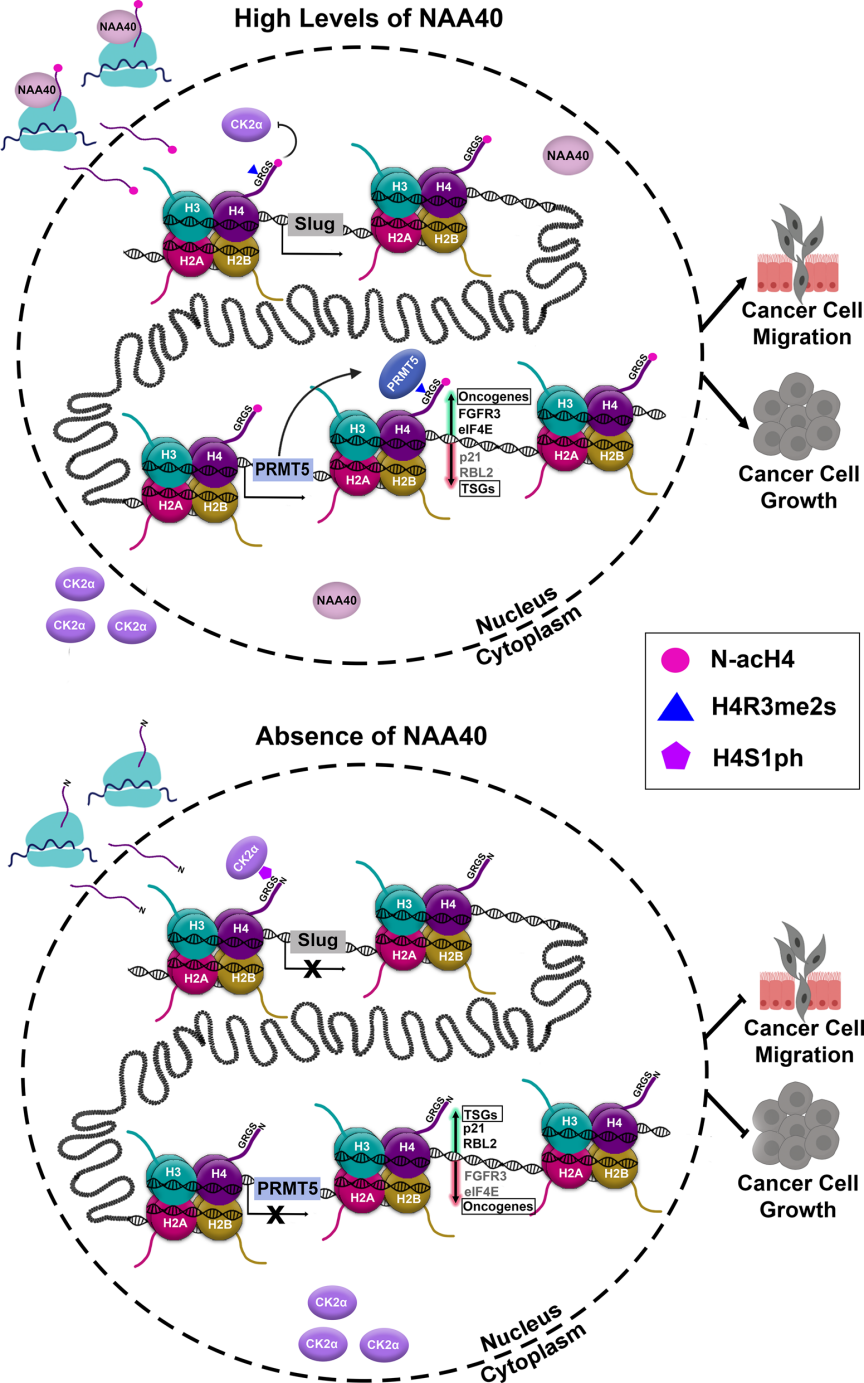
The function of NAA40-mediated N-acH4 in gene expression and cancer. NAA40 is mainly located in the cytoplasm where it binds to ribosomes and catalyzes the co-translational Nt-acetylation of histones. Although NAA40 can be also found in the nucleus, its function there remains unknown. In the presence of high levels of NAA40, N-alpha terminal acetylation on histone H4 (N-acH4) catalyzed by NAA40 blocks CK2α-mediated phosphorylation on the side chain of serine 1 residue (H4S1ph). As a result, the expression of Slug gene is activated inducing lung cancer cell migration and metastasis [28]. Additionally, NAA40-mediated N-acH4 promotes the expression of PRMT5 enzyme which then catalyzes the addition of symmetric dimethylation on the adjacent arginine 3 residue (H4R3me2s). This activates the expression of oncogenes (e.g., FGFR3 and eIF4e) while it prevents the expression of tumour suppressor genes (TSGs) (e.g., p21 and RBL2) leading to increased CRC cell growth [30]. In the absence of NAA40 and of its corresponding N-acH4, the CK2α kinase is able to accumulate in the nucleus where it binds histone H4 and catalyzes H4S1ph leading to Slug silencing and inhibition of lung cancer cell invasion. Lack of NAA40 also suppresses PRMT5 expression and thus attenuates H4R3me2s resulting in silencing of oncogenes and reactivation of TSGs delaying CRC cell growth

Evidence for the involvement of NAA40 in cancer has also been presented in recently published studies. In lung cancer cells knockdown of NAA40 was shown to reduce invasion and metastasis in vitro and in vivo. The proposed mechanism was that Nt- acetylation on serine 1 of histone H4 (N-acH4) antagonizes the CK2α-mediated phosphorylation on the side chain of the same serine residue (H4S1ph) to regulate Slug expression and metastasis [28] (Figs. 2 and 3). Recently, our group has reported that NAA40 can also affect the survival of colorectal cancer (CRC) cells. Loss of NAA40 in CRC cells triggered apoptosis through a p53-independent pathway [29]. Subsequently, in a separate study we showed that NAA40 depletion inhibits cell proliferation and survival of different CRC cell lines in vitro and in xenograft models, and increases their sensitivity to 5-Fluorouracil (5-FU) treatment. The underlying molecular mechanism was proposed to include altered deposition of symmetric dimethylation of histone H4 arginine 3 (H4R3me2s) through transcriptional regulation of the protein arginine methyltransferase 5 (PRMT5). Specifically, it was reported that lack of NAA40 and of its associated N-acH4 suppress PRMT5 expression and hence reduce the global levels of H4R3me2s resulting in altered expression of critical cancer-associated genes and the inhibition of CRC cell growth [30] (Fig. 3). An additional article reported that NAA40 is downregulated in hepatocellular carcinoma tissues compared to its highly expressed levels in normal liver specimens supporting a potential context-specific tumour suppressive property of NAA40 [31].

It is important to mention that the mechanisms through which Nt-ac of histones H4 and H2A influences gene expression are still incompletely understood. Although histone In-acetylation is a mark of active transcription [8], global reduction in H4/H2A Nt-acetylation was shown to cause both enhanced and reduced expression of specific genes depending on the molecular or cellular context [26]. As mentioned before, direct cross-talk of Nt-acetylation with H4R3 methylation and with H4S1 phosphorylation have been implicated in the regulation of gene expression and cell phenotypes [25, 28]. This raises the possibility that the effects of N-acH4/H2A are propagated through the action of specialized “reader” and “eraser” proteins. Until now, there are no known deacetylases or readers of Nt-acetylation.

Beyond the more conventional gene regulatory mechanisms employed by other histone modifications, another intriguing possibility is that changes in the global levels of histone Nt-ac can also directly influence cellular metabolic homeostasis. A compelling hypothesis put forward lately is that due to the abundance of histone proteins, the consumption of intermediate metabolites, such as Ac-CoA and SAM, during the deposition of PTMs/CTMs can influence cell metabolism [32, 33]. Given the fact that Nt-ac decorates 85–99% of histones H4/H2A [19, 20], could a significant increase or decrease in the levels of Nt-acetylation have a prominent effect on the acetyl pool of cells? It is estimated that the approximately six billion nucleotides of the human genome are wrapped around 3 × 10^^7^ nucleosome units. The potential maximum acetylation of N-acH4/H2A (100% of H2A and H4 acetylated) would therefore require close to 100 µM of Ac-CoA units (3.2 × 10^^7^ nucleosomes X 4 H2A and H4 molecules per nucleosome), an amount that is much higher than the free intracellular pool of Ac-CoA units in mammalian cells which is estimated at about 20 µM [34]. Hence, perturbations affecting the consumption of Ac-CoA by NAA40 for catalyzing Nt-ac could possibly have an impact on cellular metabolism. Nevertheless, experimental evidence for this possible link between Nt-ac and metabolic pool is not currently available.

Consequently, Nt-acetylation of H4 has emerged as a regulatory modification, with novel functions in cells that go beyond protein stability. However, further research is required to illuminate the biological and physiological relevance of N-acH4 and that of the less characterized N-acH2A.

### Histone H1 and H2B N-alpha terminal acetylation, the less studied modifications

The Nt-acetylation of the linker histone H1 is another consistently detected modification, but poorly studied and understood compared to that of H2A and H4. Among histone proteins, H1 has the largest number of distinct variants which can be immensely modified [35]. The notion that the serine 1 residue at the N-terminal tip of H1 could be acetylated was initially speculated due to a difficulty in sequencing the protein N-terminus by Edman degradation [35]. Application of MS-based proteomic methods have confirmed the presence of Nt-acetylation of H1 (N-acH1) in all examined H1 variants in human, rat, and avian cells [18, 37–41]. Both Nt-acetylated and Nt-unacetylated H1 peptides have been detected by proteomics, but different estimates of their relative abundance have been provided ranging from Nt-acetylation being predominant [36] to an even amount of the two forms [18, 37].

**Fig. 4:**
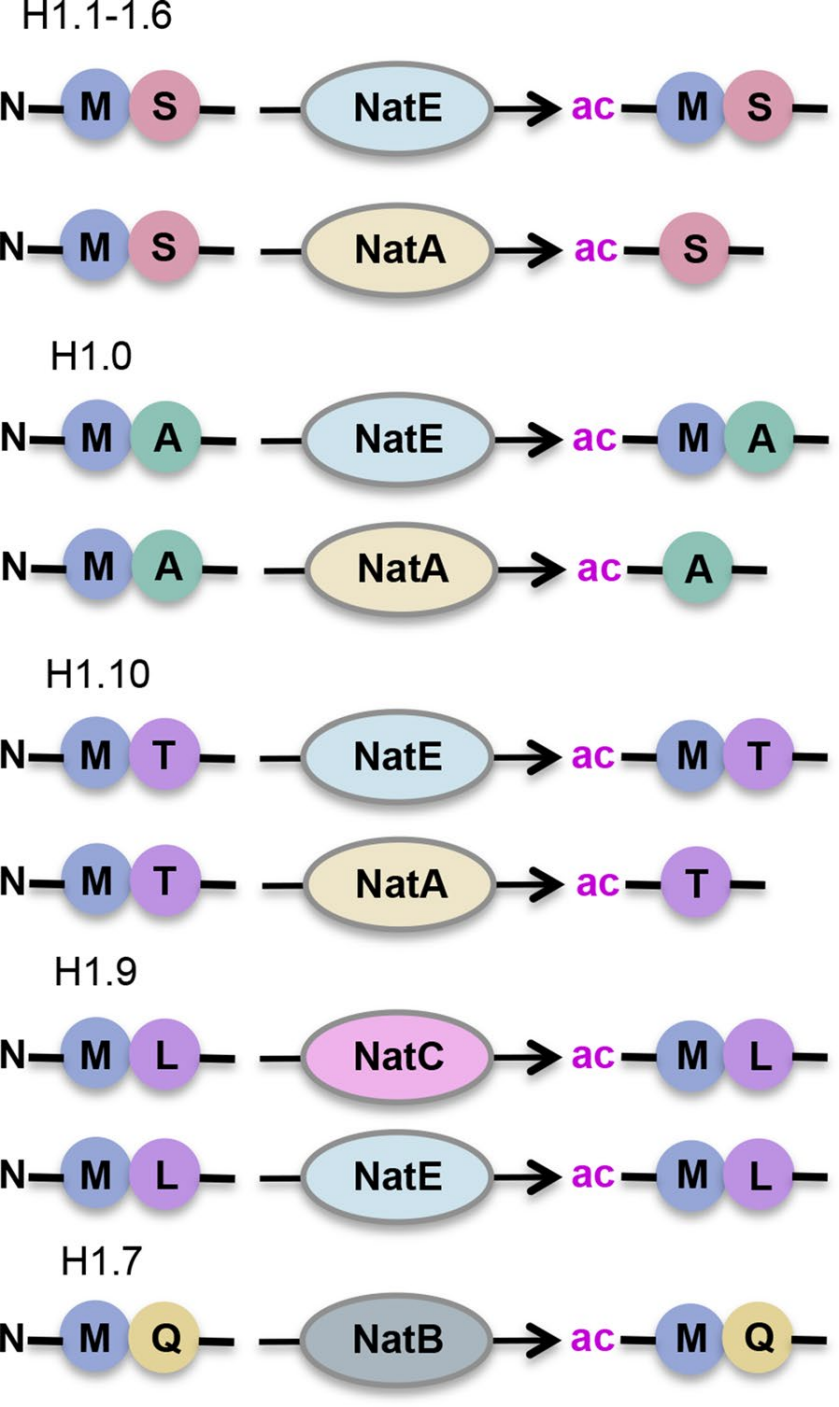
Putative N-terminal acetylation of H1 histone variants. The N-alpha terminus of initiator methionine (iMet) at H1.1-1.6, H1.0 and H1.10 variants can be acetylated by NatE. Upon excision of iMet, NatA can acetylate the N-terminal tip of serine 1 (S1), alanine 1 (A1) or threonine 1 (T1) at H1.1-1.6, H1.0 and H1.10 variants, respectively. Histone H1.9 with N-terminal sequence of iMet-Leucine 1 (L1) can be targeted by NatC or NatE while the N-terminal iMet-Glutamine 1 (Q1) 1 sequence of H1.7 can be acetylated by NatB [21]

The H1 variants differ in their N-terminal sequences, but none contains the SGRG substrate recognition motif for NAA40. They are, however, predicted to be targeted by other members of the NAT family (Fig. 4). The H1.1–1.6 variants with N-terminal end of iMet- Ser(1), H1.0 with iMet-Ala(1), and H1.10 with iMet-Thr(1) can be acetylated by NatE at methionine or NatA after removal of the initiator methionine. Histone H1.9 with N-terminal sequence of iMet-Leu(1) can be targeted by NatC or NatE, and H1.7 with N-terminal iMet-Glu(1) by NatB [21]. Whether N-acH1 is responsive to its environment is currently unknown, although interestingly Nt-ac of the H1.0 subtype has been reported to increase in aging cells [38, 39]. Nevertheless, the biological function and significance of histone H1 Nt-ac remain currently elusive but intriguing.

Partial Nt-acetylation of H2B was detected in yeast, and this modification was shown to be lost following deletion of NatA [40, 41]. How conserved this modification is and whether it is biologically active have not been examined.

## Histone N-alpha terminal methylation

N-alpha terminal methylation (Nt-me) on histone proteins is catalyzed by N-terminal methyltransferases (NTMTs). N-alpha terminal trimethylation (Nt-me3) or dimethylation (Nt-me2) generates a permanent positively charged N-terminal α-amino group, eliminating the nucleophilicity of the α-amino nitrogen. However, N-alpha terminal monomethylation (Nt-me1) changes only slightly the basicity and reactivity of the α-amino group without any severe effects.

The first eukaryotic N-alpha terminal methyltransferases, denoted as NTMT1 (also known as NRMT1; METTL11A) and NTMT2 (also known as NRMT2; METTL11B), were identified less than a decade ago [42], with the evolutionarily conserved NTMT1 currently being the only known histone Nt-methyltransferase. Following iNt-Met cleavage, the distributive NTMT1 enzyme catalyzes the mono-, di-, or trimethylation of the exposed α-amine at the first N-terminal amino acid residue of the nascent polypeptide. Although NTMT1 can modify numerous proteins with different sequence motifs [43], histone profiling studies have determined that NTMT1 recognizes the common “X(1)-Pro(2)-Lys/Arg(3)” (XPK or XPR where X signifies any small side chain amino acid) signature motif found in the human histone CENP-A (GPR) and in the fruit fly histone H2B (PPK) [44, 45]. As opposed to NATs, NTMT enzymes are mainly localized in the nucleus where they target histone proteins post-translationally.

Although demethylases targeting internal histone methylation (In-me) are well documented [46], similarly to the case of Nt-ac, no Nt-demethylases have been identified so far. A possible hint at the existence of bona fide Nt-demethylating enzymes is based on the observation that Nt-me of the non-histone MYL9 protein can be interchangeable with Nt-ac [43].

## Nt-methylation of CENP-A and its function at the centromeres

CENP-A (centromeric protein-A) is a centromere-specific histone H3 variant located in active centromeric regions of chromosomes [47]. During mitosis, CENP-A governs the formation of the constitutive centromere-associated network (CCAN) to mediate kinetochore assembly and microtubule attachment on sister chromatids [48]. Although the core domains of the canonical H3.1 and CENP-A histones are structurally very similar, their N-terminal tails are highly heterogeneous. Unlike the conventional histone H3 variant, the histone tail of CENP-A has few lysine residues, and the arginine residues appear to be mostly unmodified. Hence, the internal modifications which impact histone H3 do not appear to be as prominent for regulation of CENP-A [49, 50].

**Fig. 5:**
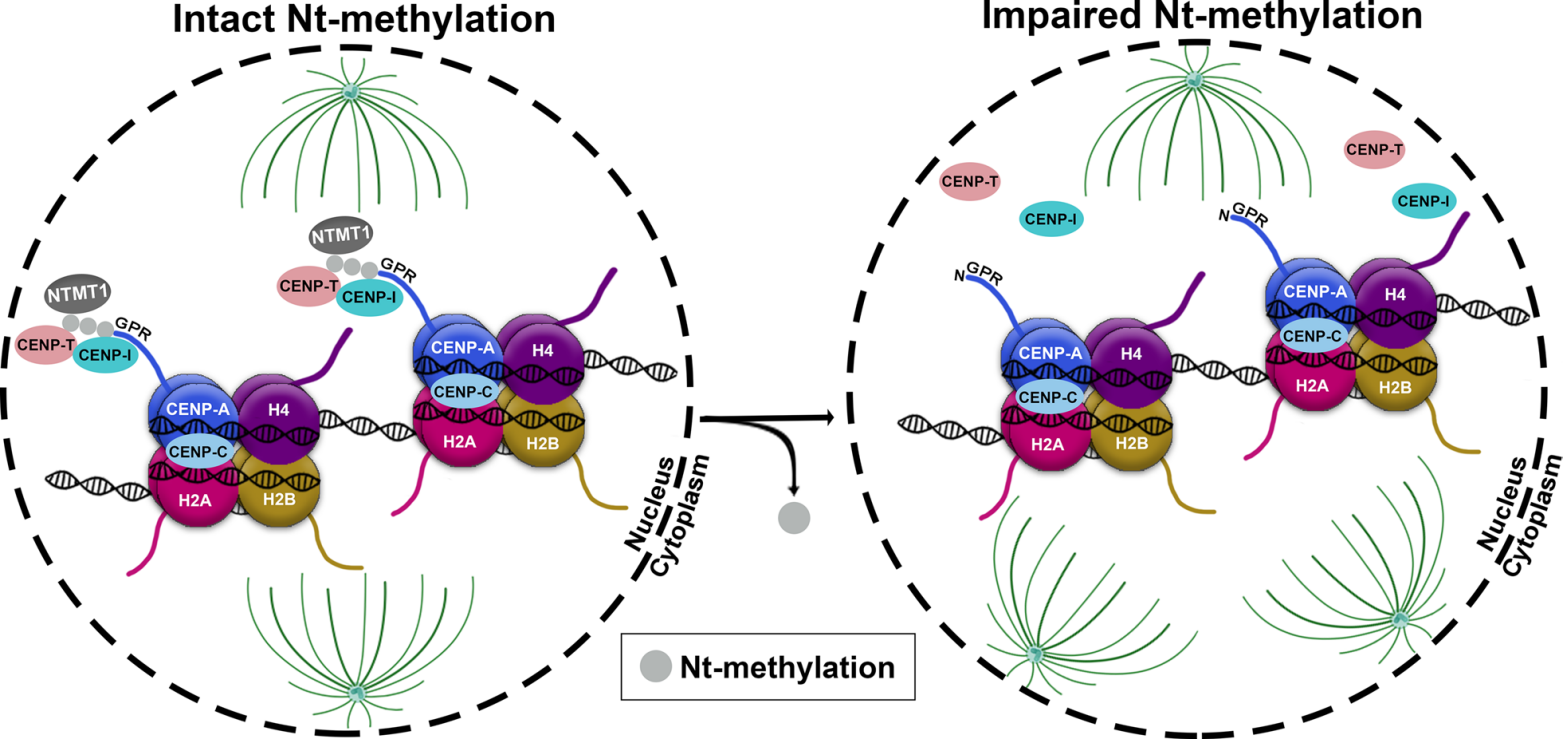
The role of NTMT1-mediated N-me [3] CENP-A in maintaining proper kinetochore formation and function. Nt-trimethylation of the centromere specific histone CENP-A (N-me [3] CENP-A) by NTMT1 methyltransferase recruits the CENP-T and CENP-I components of the CCAN complex at centromeric chromatin to secure kinetochore function and accurate segregation of chromosomes. Lack of Nt-methylation impairs the proper localization of CENP-T and CENP-I at the centromere therefore leading to chromosome missegregation and multipolar spindle formation, which has been shown to accelerate tumour formation in p53-/- colorectal cells [49]

Using affinity purification and mass spectrometry it was demonstrated that the N-terminal tip of CENP-A is methylated [49]. Upon iNt-Met removal, the α-amino group of the N-terminal glycine 1 (Gly1) amino acid of CENP-A is trimethylated by NTMT1 before nucleosome deposition of CENP-A and while it is in the prenucleosomal complex [49]. The N-terminally trimethylated CENP-A (N-me(3)CENP-A) is ubiquitous among CENP-A containing nucleosomes and is enriched with cell cycle progression, with only less than 10% of CENP-A being unmethylated [49]. One hypothesis that has been proposed regarding the importance of the Nt-methylation of CENP-A is that the addition of three methyl groups on Gly1 at the N-terminus of CENP-A could confer a permanent positive charge that enables electrostatic interaction with the negatively charged DNA. However, this hypothesis has not been proven experimentally so far. In a follow-up study, Sathyan et al. illustrated that methylation of CENP-A by NTMT1 is essential for maintaining the fidelity of chromosome segregation and proper localization of the CENP-T and CENP-I components of the CCAN in the centromeres (Fig. 5). As a result, loss of N-me(3)CENP-A by NTMT1 knockdown, CENP-A suppression or expression of a methylation defective CENP-A leads to mitotic spindle multipolarity (Fig. 5). Intriguingly, colorectal cancer cells lacking Nt-me3 of CENP-A acquire a p53-dependent growth advantage in vitro and in vivo, implying an anti-oncogenic potential of NTMT1-mediated N-me(3)CENP-A [44]. Whether similar functional consequences of NTMT1 and of its corresponding N-me(3) CENP-A exist in other tumour types remains to be explored.

### Drosophila H2B Nt-methylation and its putative role in stress response

The addition of methyl marks on the α-amino group of the N-terminal proline 1 (Pro1) residue of *Drosophila melanogaster* histone H2B (dmH2B) has also been reported [51]. Application of stress in cultured Drosophila cells, either by heat shock or arsenite treatment, triggers a significant increase in Nt-methylation levels of dmH2B which is positively correlated with transcriptional silencing. Based on this observation it was then proposed that hypermethylation at the N-terminal tip of dmH2B following stress induction gives a permanent positive charge to the α-amino group that could possibly strengthen the interaction of histone H2B with the linker DNA affecting chromatin organization and stability [51]. However, the enzyme responsible for depositing this modification was not identified by that stage.

Villar-Garea et al. first reported that dNTMT (CG1675), Drosophila’s ortholog to human NTMT1, is responsible for the mono- (N-me(1)H2B) and dimethylation (N-me(2)H2B) of the N-terminal α-amino group of dmH2B [52]. Regarding the biological function of this modification, this study showed that the levels of Nt-methylation on dmH2B increase during fly development indicating a possible regulatory role in cell proliferation, differentiation and senescence. At the molecular level, the authors of this study reported that the H3R2-specific dART8 methyltransferase physically interacts with dNTMT/CG1675 enzyme forming a complex that negatively controls the levels of Nt-methylation on dmH2B [52]. This finding suggests a potential crosstalk mechanism between H3R2 methylation and H2B Nt-methylation which may be affected by external stresses driving distinct transcriptional programs [52].

Interestingly, Nt-methylation of H2B has also been observed in invertebrate species including *Tetrahymena* [53] and *Arabidopsis thaliana* [54]. Although the H2B consensus sequence for NTMT1 is conserved from ciliates to insects, indicating a crucial biological activity of N-me(1)/(2)H2B in these organisms, the H2B N-terminal sequence deviates in mammals, and none of the multiple human H2B histone variants contain the X(1)-Pro(2)-Lys/Arg(3) NTMT1 recognition motif [48], possibly suggesting that this mechanism was evolutionarily lost in these eukaryotes.

### Could targeting the Nt-modification regulatory network provide a new therapeutic avenue?

A large body of evidence strongly supports that malfunctions in the expression or the activity of histone modifiers (writers, erasers and readers) are crucial determinants in various diseases including cancer [55, 56]. Hence, targeting epigenetic regulators is considered a promising approach in cancer therapy and has come under heavy investigation [55]. Although our understanding of the regulation and biological significance of histone Nt-modifications is far from complete, recent advances in the field advocate the potential of histone NTT, such as NATs and NTMTs, as promising therapeutic targets. The strongest case so far for an active role in cancer intervention relates to Nt-acetylation on histones H4 and H2A mediated through NAA40. Of particular interest is a recent study examining the prevalence of histone mutations across a wide range of cancers. It was reported that specific substitution of serine 1 to cysteine (S1C) is the second most frequent mutation on histone H2A and the most common on histone H4 proposing that these mutant forms may represent common oncohistones [57]. Whether these mutations affect the abundance of Nt-acetylation on this first histone residue is not known and could be explored in future studies. In addition, as we discussed before, NAA40 has been reported to be required for the survival and invasion abilities of colorectal and lung cancer cells, respectively [28, 30], highlighting its potential as a therapeutic target.

Several observations support the possibility that reversing aberrant H4/H2A Nt- acetylation in cancer by targeting NAA40 may be an attractive therapeutic strategy. First, NAA40 has been shown to have oncogenic functions in at least two cancer types (lung and colon), while its expression is increased in several other cancer types (TCGA data https://portal.gdc.cancer.gov/). Second, therapeutic silencing of NAA40 potentially could have few side effects because, in contrast to the majority of epigenetic modifiers, it is highly selective known to target only two substrates (histones H2A and H4) and functions as a monomer [17]. Therefore, it is expected that manipulating NAA40 activity will have less off-target effects compared to other epigenetic modifiers, which act on a large number of substrates. Additionally, no detrimental effects of targeting NAA40 were observed in non-cancer mouse embryonic fibroblasts [29], thus it is possible that inhibiting this histone modifier will not affect adversely normal cells. Third, NATs can be targeted by specific chemical inhibitors, as has been shown with the previous design of selective bisubstrate analogues against NatA, NAA10, and NAA50 [58]. Finally, the recent elucidation of the protein structure of NAA40 revealed unique features compared to other related NAT proteins, which could allow its selected targeting without affecting other members of its family [23]. Based on these properties, NAA40 is a potentially druggable enzyme that could be exploited in anti-cancer therapy.

NTMT1 has also been implicated in carcinogenesis, but whether it acts as a tumour-suppressor or an oncogene depends on the pathways driving cancer formation in the given tissue. Mutations which impact the enzymatic activity of NTMT1 have been detected in cancer. Experimentation on two NTMT1 mutants, N209I (found in endometrial cancer) and P211S (found in lung cancer), revealed that these mutants yield reduced Nt-trimethylase and increased Nt-monomethylase/dimethylase activity [59]. The robust upregulation of NTMT1 that has been reported in different cancer types and its significance in promoting cell proliferation suggest a role of NTMT1 as an oncoprotein [60]. Conversely, other studies display evidence pointing towards a tumour suppressive function of NTMT1. For instance, in breast cancer cells loss of NTMT1 promotes marks of oncogenesis (proliferation, invasive potential), but also increases sensitivity to DNA damaging agents [61].

The latest discoveries on the structural basis of NTMT1-mediated Nt-methylation together with its previously reported oncogenic function encouraged efforts to design and synthesize potent and specific inhibitors of the enzyme [62]. Recently, bisubstrate inhibitors with a high potency and specificity against NTMT1 were reported [63]. However, careful interpretation is required when using such developed NTMT1 inhibitors because this enzyme targets numerous substrates beyond histone proteins [64].

## Conclusion

As described in this review, histone Nt-modifications form a distinct category from Internal histone marks and they are mediated by a different group of enzymes. The emerging evidence suggesting that these modifications are not biologically inert has motivated more intense and focused research on their associated molecular mechanisms and biological roles. The currently known histone Nt-modifications are acetylation of histones H1, H2A, H2B, and H4 and the methylation of human CENP-A and invertebrate H2B (Table 1). It is interesting to note that Nt-acetylation and methylation appear to impact different histone proteins, with no evidence of overlap or competition. This fact could reflect their independent evolution, with no commonalities in their regulations or biological functions. In the case of histone Nt-ac, this CTM has in recent years been implicated in transcriptional activation, conveying environmental signals and controlling the expression of specific genetic pathways. A fundamental mechanism through which Internal PTMs define gene expression patterns is by cross-regulating each other [65]. The work of different groups, which is summarized in this review, strongly supports that Nt-modifications control transcription through their cross-talk with distinct In-marks (Fig. 2). Yet, despite the recent insights in the field future research is necessary to unlock and verify the functional readout of Nt-modifications on histone proteins. These studies should enable a deeper understanding of why these modifications are so strongly evolutionary conserved, as well as uncover their full therapeutic potential.

**Table 1:** List of known histone N-alpha terminal modifications and their associated NTTs

Histone	Species	Enzyme	Recognition motif	Biological function(s)	References
Nt-acetylation					
H1	Detected in mouse and human cells	Potentially various NATs	Various	Not known	[18, 68, 69]
H4/H2A	Conserved from yeast to human	NAA40	SGRG	Response to caloric restriction; role in carcinogenesis	[26] [30]
H2B	Yeast	NAA10	S	Not known	[40, 41]
Nt-methylation					
CENP-A (H3 variant)	Mammalian species	NTMT1	GPR	Formation of CCAN	[49, 50]
H2B	Invertebrate species from ciliates to insects	NTMT1	PPK	Stress response	[51]

An urgent challenge is to determine whether specific bromodomain and chromodomain-containing “Nt-reader” proteins exist that recognize, bind and interpret Nt-ac and Nt-me, respectively. After all, In-modifications very frequently function by recruiting or occluding other regulators, such as chromatin remodelers and transcriptional regulators, on chromatin. It is therefore possible that Nt-modifications also function in such a manner. Experimental methods such as those used to find In-readers (e.g., SILAC, pull-downs, etc.) could also be applicable to these cases. Then again, given the very high abundance of Nt-modifications on histones compared to In-modifications, it is also possible that they work through distinct mechanisms rather than by conventional readers. For instance, they may regulate the activation or silencing of gene expression by controlling the metabolic status of the cells [32]. Furthermore, the marks on the tip of histone tails have been characterized as stable in contrast to the dynamic internal PTMs, raising one of the most challenging questions in the field: Are they truly irreversible? Taking lessons from demethylases of In-me it is very plausible that in the near future researchers working in this area may identify currently unknown N-alpha terminal de-modifying enzymes [66]. Finally, future proteomic studies could unveil the entire repertoire of histone Nt-modifications. For example, other histone Nt-acylations potentially exist such as formylation and propionylation since these modifications have been observed on other protein N-termini [67]. Altogether these emerging areas of interest will further strengthen our knowledge regarding histone Nt-modifications, opening new horizons for these ancient modifications.

## Data Availability

All data generated or analyzed during this study are included in this published article.

## Abbreviations

Ac-CoA: Acetyl-coenzyme A
CENP-A: Centromeric protein-A
CTM: Co-translational modification
HAT: Histone acetyltransferase
HDAC: Histone deacetylases
In: Internal
In-ac: Internal acetylation
In-me: Internal methylation
KDM: Lysine demethylase
KMT: Lysine methyltransferase
MS: Mass spectrometry
NAA40: N-alpha-acetyltransferase 40
NATs: N-terminal acetyltransferases
Nt: N-alpha terminal
Nt-ac: Nt-acetylation
N-acH2A: Nt-acetylation of histone H2A
N-acH4: Nt-acetylation of histone H4
Nt-me: Nt-methylation
NDAC: N-terminal deacetylase
NTDM: N-terminal demethylase
NTMT: N-terminal methyltransferase
NTT: N-terminal transferases
PTM: Post-translational modifications
PRMT: Protein arginine methyltransferase
SAM: S-Adenosyl methionine
